# Effect of Covid-19 pandemic on tourist travel risk and management perceptions

**DOI:** 10.1371/journal.pone.0256486

**Published:** 2021-09-01

**Authors:** Muhammad Khalilur Rahman, Md. Abu Issa Gazi, Miraj Ahmed Bhuiyan, Md. Atikur Rahaman

**Affiliations:** 1 Faculty of Entrepreneurship and Business, Universiti Malaysia Kelantan, Pengkalan Chepa, Malaysia; 2 School of E-Commerce, Jiujiang University, Jujiang, Jiangxi, China; 3 School of Economics, Guangdong University of Finance and Economics, Guangzhou, China; 4 School of Management, Jiujiang University, Jujiang, Jiangxi, China; Institute for Advanced Sustainability Studies, GERMANY

## Abstract

This study aims to explore the impact of the Covid-19 pandemic on tourists’ travel risk and management perceptions. Driven on the effect of the pandemic, we investigate tourists’ travel risk and management perceptions and its effect on society using a sample of 716 respondents. The data was collected through social media platforms using a representative sampling method and analyzed applying the PLS-SEM tool. The findings reveal that Covid-19 pandemic has greatly affected travel risk and management perceptions. Travel risk and management perception had a significant association with risk management, service delivery, transportation patterns, distribution channels, avoidance of overpopulated destinations, and hygiene and safety. The results also identified the mediating effect of travel risk and management perceptions. The finding of this study contributes to tourism crises and provides future research insights in the travel and tourism sector and response to change tourists’ travel risk and management perceptions in the post-covid recovery period.

## Introduction

The world tourism industry is facing the effect of the Covid-19 pandemic. Tourists’ travel risk and management perceptions are crucial matter in their decision to travel destinations during the ongoing uncertainty of Covid-19 epidemic. Tourists’ travel risk and management perceptions can influence their psychological behavior for travel to destinations [1, 2] Tourists can view their travel risk and management issues differently due to the spread of the existing pandemic. Tourists will avoid visiting destinations if they consider it risky [3]. Tourists’ travel risk and management are associated with tourism destinations, which is multidimensional where the outcomes are uncertain due to the impact of Covid-19. Therefore, it is difficult to recognize the common risk and management dimensions for developing a theoretical foundation based on the tourists’ risk and management perceptions and incorporating their outcomes. However, due to having a crucial concept of travel risk during the Covid-19 pandemic, this study has paid attention to explore and evaluate the tourists travel risk and management perceptions associated with the tourism attractions.

The Covid-19 pandemic has ruined all the previous narratives on development. Lockdowns at the largest scale in human history have imposed by governments around the world to control the spread of the pandemic. The consequences of this pandemic could change many aspects of human life and business including tourism management as almost half of the global population adopted restrictions on movement at an unprecedented scale. The Covid-19 is an infectious disease caused by a new strain of coronavirus. Co stands for corona, Vi for a virus, and D for the disease. This disease refers to as 2019 novel coronavirus or 2019-nCoV. The impact of the novel Covid-19 pandemic is expected to have antagonistic results on the tourism sector, and the economy worldwide [4]. The economic estimations are foreseeing diminished financial development and showing negative attitudes to residents from countries most intensely affected by the Covid-19 pandemic [5]. The Covid-19 pandemic started at Wuhan in China in December 2019 [6, 7] and other countries in February 2020. It has various effects and countries around the world are looking for a sustainable development approach to mitigate its negative impact. The pandemic is calamitous for recovering the economy of every country, nonexistent the travel industry, and social angles including long-term health issues in those affected by the infection and losses the friends and family. The effect of Covid-19 has mental effects [8] and it appears to be essential to identify them appropriately and address these issues to directly control the spread of infection [6].

Societal wellbeing or safety measures through lockdowns can control the spreading of infections [5]. However, when such safety measures are excessively strict, they can have negative impacts on developing the tourism industry, interruption of economic development, and increase the unemployment rate. It is reported that the business world today is directly or indirectly impacted by different external factors such as financial, sociocultural, global, political, and technological [4]. The changes in these factors lead to a change in business performance in industry in the region-specific or worldwide. The world is aware of the Covid-19 pandemic and its social outcomes remain ambiguous [9]. Although China, the United States and other developed countries have produced vaccines and started vaccination, most of the developing countries are struggling for getting the vaccine for protection against the outbreak of the Covid-19 epidemic. There is a lack of healthcare safety and security in many countries regarding handling Covid-19 patients, lack of doctors, a lacuna of vaccine, and testing facility. Covid-19 is a global phenomenon, and it may appear soon as an established external factor in curricula on strategic management for business performance and emerging tourism marketing.

Other factors are mostly controllable by social frameworks, and individuals [4]. Pandemics are generally uncontrollable because they appear suddenly everywhere. The travel and tourism sector are particularly motivated by changes in external factors and given the idea of political and financial systems. The travel industry involves various sectors and contributes to these areas’ advancement and the global value of tourism management. The effect of the Covid-19 pandemic on the tourism destination, tourists’ behavior, and their preference is irrespective of district or nationality. The earlier studies [9, 10] have confined the connection between pandemic and tourism regarding risk. Few studies [11] analyzed the tourism restrictions on the spread of the Covid-19 pandemic and explained how destinations decided to react to a pandemic. Travel and tourism are one of the largest industries all over the world [12, 13], however, despite this industry, the hospitality and tourism industry is currently highly sensitive to significant shocks (e.g. Covid-19 pandemic). It is crucial to investigate how the tourism industry will recover from the effect of the Covid-19 pandemic.

The rapid transmission and high mortality rate of the Covid-19 pandemic lead to the scientific community monitoring its spread of infection [14]. The pandemic encourages the continuation of social quarantine and adverse financial effects. The clinicians and researchers have expressed their concerns about the negative effects of the Covid-19 epidemic on the health of people and behaviors [15]. Recently a few studies discussed Covid-19 from healthcare perspectives [5, 8]. Some studies focus on the risk management of the Covid-19 pandemic [16, 17]. Some researchers [18] focus on the travel and tourism crisis while others [10] proposed the necessary procedures that prevent potential biosecurity threats because of worldwide pandemic outbreaks. There is a study that [19] focused on the Covid-19 pandemic and its effect on Chinese residents’ lifestyle and travel, which leads to enlightening long-term patterns of behavior and tourism destination. A few countries have made explicit strides in suspending their visa on arrival strategy and initiating strict travel bans to control the spread of the pandemic. Another research study [20] reported that the Covid-19 epidemic has carried economic collapse to Singapore, Bali, Barcelona, Rome, and other counties that were once tourists’ attractions. The effects of this outbreak on the travel and tourism industry in the world have been extremely debated by industry practitioners, the tourism department of the government, and the academic community.

Most of the countries all over the world are decided to close their borders and postpone their airline’s services due to the Covid-19 pandemic. United Nations World Tourism Organization reported that there is a global crisis in the tourism industry and Covid-19 is responsible for a decline of international tourist arrivals that estimate the losses of US$300–450 billion [19]. This is surprisingly more terrible than the effect of SARS in 2003 [21]. The Covid-19 pandemic has affected many countries and the global tourism industry faces terrible situations in which business has been closed, lives have been lost, and people are on high alert for social safety. The earlier studies [8, 9, 22, 23] indicate that the academic community timely provides research for everyone’s benefit over the healthcare, sociologies, and hard science. Concerning this research, the existing study aims to investigate the social impact of the Covid-19 epidemic on tourism destination and tourists’ behaviors as well as their preferences during this pandemic. This investigation likewise explains how global travel and hospitality practices are probably going to change because of the pandemic. This study depends on the synthesis of early literature and sources of published news and reports related to tourism management, marketing, healthcare, and tourist behavior. Based on these, the study draws a conceptual model for empirical assessment. For the post-Covid-19 and business recovery, these insights will assist tourism operators, managers, marketers, and industry practitioners tailor their tourism products and services.

## Literature review

### Underpinning theory

This study uses the concept of pathogen-stress theory [24] to evaluate the travel risk and management perception due to the Covid-19 uncertainty and determining human behaviors in societal issues. Some authors have [25] explored the influence of pathogen thereat in the context of Covid-19 epidemics. The personality traits are predicted by a parasite-stress theory of human sociality that highlights the infection risks related to the interaction with conspecifics [24, 26]. The travel risk and management perception refer to the risk of human-to-human transmission. The infection risks are connected to the openness of human contact. The increased contact with many group members implies a higher risk of human-to-human transmission. According to this theory, when people develop in a parasite-infested environment, they become less open to visitors, less curious, less exploratory and reduce their chance of infection. This theory is not only emphasized cultural differences but also cultural difference over space such as between different human populations. Generalizing the concept of pathogen-stress theory, this study explores the effect of Covid-19 epidemic and its impact on travel risk and management perceptions.

### Effect of Covid-19 pandemic

Covid-19 is a new pandemic that first erupted in December 2019 in China and spreads rapidly across the world through human-to-human transmission. Most countries all over the world are instituting short-term travel restrictions to stop the spread of infection which increase the concern caused by the Covid-19 pandemic on the tourism industry worldwide [5]. Researchers must think about the previous disaster of the 2003 SARS outbreak [27] and the 2004 tsunami in Sri Lanka [28] for lessons on how to manage the crisis from the disaster [19]. Tourists prefer an inclusive tourism package, safety and security when travelling to popular destinations. They want to avoid risk and crowded tourism destinations, and they may decide not to visit destinations if their destination preferences diminished well-being after the outbreak. The covid-19 pandemic is already brought severe concerns to the world tourism industry and niche market. United Nation [21] reports that the recent circumstance of the tourism sector is very worse due to the pandemic. This crisis expanded in the world and Covid-19 pandemic easily immobilize international tourists’ emotional stability. The impact of Covid-19 epidemic is greatly affected tourists’ travel risk and management perception. Researchers [19] suggested the practitioners for exploring the tourists’ travel behavior towards tourism destinations. The discussion of existing literature evidence that there is no empirical examination that focuses on the impact of Covid-19 pandemic on tourists’ travel risk and management perception. Thus, we propose the hypothesis:

H1. The fear of Covid-19 pandemic affects the tourists’ travel risk and management perception.

### Tourists’ travel risk and management perception

Travel risk and management perception refer to the evaluation of a situation concerning the risk to make travel decisions in destinations [1]. Travellers’ risk and management perception is a key component for tourism destinations. Risk management refers to the practice of recognizing potential risks of the travel and tourism industry due to the current pandemic in analyzing, improvement and taking preventive steps to reduce the risk. Many countries of the world started to recover from the crisis of tourism events [2]. Tourists’ travel arrangement should be organized to minimize the risk and stress of tourists. For example, tourists should purchase insurance when they booked trips to destinations. Researchers [29] stated that the travel and tourism industry is vulnerable against risk including crises events, epidemics, pandemics, and other risks that challenges tourists’ safety. The previous studies indicated that risk restricts travel is negatively affect tourism demand [30–32]. Other authors [33] found that perceived risk negatively affects tourists’ destination perceptions. This study postulated that:

H2. Tourists’ travel risk and management perception have a significant impact on risk management.

Travel risk indicates the cancellation of flights due to the tourists’ travel restrictions, travel risk and management perceptions. The travel cancellation leads to tourists’ negative emotion, anxiety and disappointment [34]. In line with this, service delivery or service efficiency is crucial to tourism initiative performance. Service failure could lead to a negative impact on travel destinations. The previous studies indicated that tourists’ travel risk and management perception may negatively influence tourists’ decision making [35, 36]. Professional service delivery and timely response could reduce tourists’ travel risk and management perceptions. Studies [36] identified that some restaurant refused to provide service delivery to Chinese people. This racial discrimination may lead to tourists’ having an increase in travel risk and management perceptions towards destinations. Research study [4] stated that public health crisis can affect tourists’ dining behavior. Thus, tourist should avoid eating in restaurants and order delivery to minimize social interaction and avoid unnecessary contact with people during the pandemic. Therefore, this study postulated that:

H3. Tourists’ travel risk and management perception have a significant relationship with service delivery.

The travel behavior of people changes at the individual level due to the Covid-19 pandemic in the globe [37]. It is difficult to change the transportation pattern in the public areas and crowded public transits in the country. Articles [4] reported that bike or ride-sharing services could be alternative to more crowded transit options in the wake of Covid-19 pandemic. Social distance is important to avoid crowded areas, thus, the availability of different transportation options within the country can help tourists to decide to visit their desired tourism places. Another study [38] stated that the transportation network is vulnerable to disturbance due to movement restrictions. Research work [39] indicated that the use of public transport signifies a higher risk of infection of Covid-19 in Budapest. This study proposed the following hypothesis:

H4. Tourists’ travel risk and management perception are positively related to travel pattern.

The distribution channel refers to the traditional travel agencies to online agents while purchasing tour packages, booking hotels and buying ticket [4]. Distribution channels are the intermediaries through which a product and services pass to the end customers. Authors [40] stated that customer behavior has a significant link with purchase behavior, destination choice, experience sharing, and information searches. Information technology can easily reduce an individual’s travel risk and management in person-to-person communication [41]. For instance, people can work at home without travelling to the office, involve with distance learning, order products and services online, and performing banking transaction virtually. People use technology for travel-related purposes such as booking holidays, offering instant vendor feedback, and comparing travel destinations, which lead to reducing travel risk and management perceptions. Therefore, we proposed that:

H5. Tourists’ travel risk and management perception have a significant influence on distribution channels.

Covid-19 spreads through human-to-human transmission, thus, it is crucial to avoid overpopulated destinations. Overpopulated destination refers to the neologism that indicates the overcrowded people on a holiday destination. A collaborative work [42] indicated that pathogen threats make people alert and avoid overpopulated destination. This tendency will initiate a mind shift in people travel behavior and reduce the tourists’ travel risk and management perception in the avoidance of overpopulated destination [43]. It’s reported that social distancing can assist to prevent infection of Covid-19 epidemics [44]. According to several studies [4, 45, 46] tourism locations are plagued by overcrowded travelers, thus, tourism operators can identify how the best way to manage tourist flows to make sure safety, well-being and risk perception of visitors. This study proposes that:

H6. Tourists’ travel risk and management perception have a significant impact on the avoidance of overpopulated destinations during Covid-19 pandemic.

The Covid-19 pandemic has made people conscious of hygiene and safety. People are concerned about their safety and hygienic need in public transports, hotels and recreational sites [47]. To reduce the symptom of people of Covid-19 epidemics, face masks use can be helpful for the hygiene and safety of people [4, 48]. Covid-19 pandemic have greatly affected the travel decision of tourists and their health safety and hygiene [4]. It implies that safety and hygiene can be a significant factor for the travel risk and management perception of tourists. Because the risk mostly belongs to safety and hygienic including health-related issues. The potential tourists are generally like to seek destinations’ safety and hygiene, cleanliness, established infrastructure, and high-quality medical facilities during the Covid-19 pandemic [4]. Thus, this study postulated that:

H7. Tourists’ travel risk and management perception have a significant impact on destinations’ hygiene and safety.

**Fig 1:**
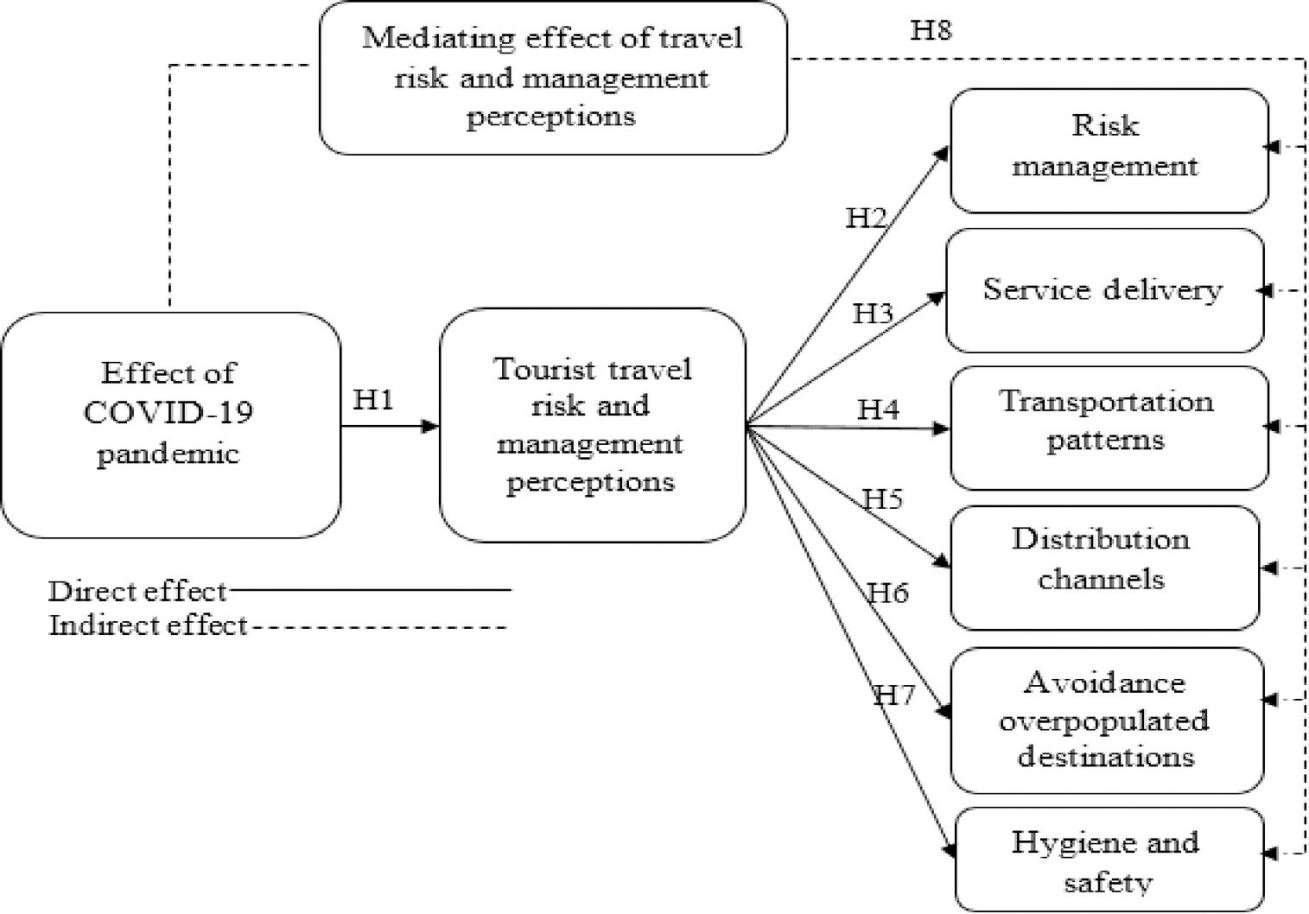
Conceptual framework.

Based on the existing theoretical and empirical assessment, this study proposes a conceptual model (**Fig 1**).

### Methodology

#### Survey instrument

This study uses an explicit statement for measuring respondents’ responses to the given factors of Covid-19 epidemic, tourists’ travel risk and management perceptions and their social traits. Studies [49] supported that this method is suitable for the respondents for an understanding of the survey measurement items. This study uses multi-measurement items for all constructs due to overcoming the limitations of using a single item. Specifically, five measurement items were modified from [8] and [19] for evaluating the effect of Covid-19 pandemic. A total of four questions measuring travel risk and management perception were adapted from previous studies [19, 50]. The five measurement items used to evaluate risk management considering tourists’ travel risk management perception to visit the destinations were modified from [5] and [19], while the three questions related to service delivery were adapted from [19]. Three measurement items referring to [19] were designed to evaluate transportation patterns, and three questions based on [41, 51] and [19] measured to assess distribution channels. Four items were modified from [44] and [4] to measure the avoidance of overpopulated destinations, while four items developed from [4, 48] to evaluate hygiene and safety. All measurement items under the constructs were assessed using a seven-point Likert scale from strongly disagree (1) to strongly agree (7).

### Survey administration and sample

The data were collected from a self-administered questionnaire to examine the conceptual model of this study. The questionnaire of this study was pretested to certify the validity of the survey instrument. To ensure content validity, the researchers of this study conducted a pilot test among 50 international tourists. The reliability test was employed to identify Cronbach’s alpha value (above 0.70) of all constructs and confirm the reliability of the survey questions [52]. In this study, an English version questionnaire was used for data collection as most of the participants were educated, and they were able to answer the survey questions. The questionnaire was delivered through an online survey using the Google platform tools and highlighted the main purpose of this study. We described the procedure of the survey to the respondents before participating in this study. The researchers of this study politely requested respondents through the online platform, explained the purpose of the study and asked for their consent to be part of participants in this study.

We ensured to the respondents that the data would be collected for academic only and no other authorities would have access to this information. Also, we confirmed to the respondents that they would remain anonymous because participants were not required to provide their name, address and mobile numbers. The survey questionnaire of the Google platform link was shared on social media (Facebook, WhatsApp, BiP) for collecting data. Also, the researchers of this study collected an email address from the respondents through Linkedin and sent them a Google platform link to the survey questionnaire. The online questionnaires could be completed with the use of respondents’ smartphone, laptop/computer. The complete survey questionnaire consisted of 63 items and they took approximately 20 minutes to complete. We adopted the cross-sectional design and collected data from 731 international tourists via an online survey from the 2nd week of April to the 1st week of July 2020. Before collecting the data, an ethical research approval letter was obtained from the Jiujiang University Research Ethics Committee (JUREC). An introductory letter and consent form was also obtained from the ethics committee, which clearly expressed the reason for this study to acquire consent from the respondents for conducting the study. Online survey approach was used for collecting data from the respondents. We sent a consent form to the respondents whether they are willing to participate in this study. The respondents of this study are individual tourists who visited different tourism destinations around the globe. In line with this, we used a representative sampling method for collecting data from the different geographic areas such as Middle East, Asia, Africa, Australia, Europe, and America. A representative sample can cover a part of the population and allows to approximate the entire population. Studies [53] indicated that a representative sample can accurately reflect the characteristics of the large group.

A total of 1000 questionnaires with consent form were sent using a Google platform and 731 were returned, confirming a return rate of 73.1%. A total of 1000 questionnaires with consent form were sent using a Google platform and 731 were returned, confirming a return rate of 73.1%. Fifteen returned questionnaires were found to have only partially completed and thus they were not usable. The usable response rate was approximately 71.6%. The respondents’ answers to the open-ended question were hand-coded and checked by the researchers of this study. In this study, the minimum sample size was according to prior power calculation. We considered recruiting at 716 respondents because this would provide satisfactory power 0.80 to detect expected correction coefficient 0.20 (https://www.sample-size.net/correlation-sample-size/). We considered a large sample size in this study since this could increase the statistical power for detecting poor effects and strengthen the robustness of the results.

### Data analysis method

In this study, we have used SmartPLS3.0 software for testing the hypothesis relationship among the indicators. The partial least square (PLS) method is a more appropriate statistical technique since it can prevent specification errors and improve the reliability of the results, as well as provide better outcomes and minimize structural errors [54]. This method is suitable for examining the hypothesis relationships of the study [55]. The PLS method consists of 2 steps, for example, measurement model and structural model [56], which has been analyzed in this study.

### Multivariate normality and common method variance

Structural equation modeling using the partial least square method is not related to multivariate normality in data, because it is a non-parametric assessment instrument [57]. It is [58] suggested that multivariate data normality can be tested using the online tool of web power (https://webpower.psychstat.org/wiki/tools/index) to estimate data normality. We run the web power and the result revealed that the data set is not normal because [59] multivariate coefficient p-values were less than 0.05 [60, 61]. In social science study, common method variance is normal due to the data collection procedures. We run [62] one-factor test [63] to evaluate the effect of common method variance on the constructs of the study. The result of one-factor Harman’s test revealed that common method variance is not a critical matter in this study because the main factor explained 33.45% variance, indicating less than the suggested limit of 50% [64].

## Data analysis

### Demographic characteristics

The majority of the respondents consisted of male (66.7) whereas female was 33.3%. In terms of the marital status of the respondents, 59.9% was married followed by a single (36.8%) and divorced (3.2%). The majority of the respondents had a bachelor’s degree (57.1%) followed by a master’s degree (24.4%), a secondary school/diploma degree 14.0, and a PhD (4.5%). The results indicated that around 87.5% of respondents were not infected by the affected Covid-19 pandemic whereas 1.2% were infected by Covid-19 and 11.3% of respondent do not know whether they were infected by Covid-19 or not. In terms of travel purpose, the majority of the respondents (39.1%) travel for leisure/holiday or shopping purposes, which followed by education/conference (28.2%), healthcare (17.0%), others (11.3%) and business (4.4%). The following are the percentage of age group: between 18–29 years old (42.0%), between 30–39 years old (33.6%), between 50–59 years old (6.7%), and above 60 years old (1.7%). The majority of the respondents were a private employee (59.9%) followed by a government employee (30.8%), and unemployed (9.2%). The following are the percentage for monthly income of the respondents: less than USD2000 (74.4%), between USD2001- USD5000 (18%), between USD5001- USD7000 (4.3%), between USD7001- USD10000 (1.6%), and above USD10000 monthly income. The majority of the respondents in this study were from Middle East (37.2%), followed by Asia (29.3%), Africa (14.1%), Australia (9.3%), Europe (7.0%), and America (3.1%).

### Measurement model analysis

In this study, we examined two types of validity such as convergent validity and discriminant validity to evaluate the measurement model. The convergent validity is assessed with two major coefficients such as composite reliability (CR) and average variance extracted (AVE). To measure the convergent validity, the factor loading of each construct should be considered and compared to a threshold. Studies [55] reported that the loading should be greater than 0.70 to measure convergent validity. Researcher [56] postulated that the items of each factor loading lower than 0.40 is required to consider for elimination. The findings revealed that the majority of the indicator loadings on their corresponding latent variables are greater than 0.80 (Table 1), indicating a higher convergent validity of the model. The CR coefficient was used to measure the construct reliability. The result showed that the value exceeded 0.80 for all latent variables, which indicates the acceptable construct reliability. The results of AVE of all latent variables exceeds the threshold of 0.50 [56], which signifies that the convergent validity of the measurement model is acceptable. The Cronbach’s alpha value exceeded the cut-off point 0.70 [54], which recognizing that internal reliability attains the acceptable level. The rho-A value exceeded that threshold 0.70 and the variance inflation factor (VIF) sowed lower than 3.3, which indicating that there is no multicollinearity issue in the model.

**Table 1:** Convergent validity.

Constructs and Items	Factor loadings	VIF	α	rho_A	CR	AVE
***Covid-19 pandemic***			0.887	0.890	0.917	0.690
I feel symptoms of infection by the Covid-19 pandemic (eco1)	0.813	2.445				
Covid-19 pandemic affect my travel/shopping behavior (eco2)	0.871	2.006				
I feel apprehensive due to the Covid-19 pandemic (eco3)	0.830	2.196				
I feel financial stress due to the Covid-19 pandemic (eco4)	0.842	2.274				
I feel stress from your work due to the Covid-19 pandemic (eco5)	0.795	1.852				
***Travel risk and management perception***			0.898	0.899	0.924	0.710
The effect of the Covid-19 pandemic has created international anxiety for travelling destinations (trmp1)	0.830	2.165				
I prefer to spend my leisure time alone due to the Covid-19 pandemic (trmp2)	0.861	2.593				
After Covid-19, I prefer to avoid travelling to crowded big cities (trmp3)	0.862	2.538				
Covid-19 reduces the possibility of travelling with groups (trmp4)	0.824	2.155				
***Risk Management***			0.884	0.885	0.915	0.683
My biggest concern about Covid-19 and how long I will be able to handle isolation (rm1)	0.820	2.018				
Many people underestimate the disease and its effect on some people (rm2)	0.819	2.093				
I wonder whether the government is providing us with all the available information about the Covid-19 pandemic (rm3)	0.826	2.292				
I seek destinations with established infrastructure following the Covid-19 pandemic (rm4)	0.837	2.356				
I seek destinations with established high-quality medical facilities following the Covid-19 epidemic (rm5)	0.828	2.081				
***Service Delivery***			0.854	0.854	0.911	0.774
I prefer to order takeout rather than eating or drinking in restaurants to avoid unnecessary contact with others (sd1)	0.892	2.318				
During this pandemic, I order delivery of my necessary things to minimize interpersonal interaction (sd2)	0.888	2.295				
I prefer the provision of packed and sanitized food (sd3)	0.858	1.873				
***Transportation Patterns***			0.845	0.845	0.906	0.763
I avoid crowded public transits (tp1)	0.870	2.006				
I prefer to use public transportation (tp2)	0.883	2.176				
I believe bike or ride-sharing services are suitable alternatives to avoid more crowded transit options in the wake of COVID-19 (tp3)	0.867	1.930				
***Distribution Channels***			0.785	0.790	0.874	0.698
I prefer online platforms while purchasing tickets, booking hotels and buying tour package (dc1)	0.815	1.638				
I think online platforms are suitable for information searches, destination choice, and purchase behavior, and experience sharing (dc2).	0.841	1.571				
Using the distribution channels, people can work from home and engage in social distance learning (dc3)	0.850	1.725				
***Avoidance of Overpopulated destinations***			0.803	0.810	0.883	0.716
I avoid unnecessary interaction with crowds in public spaces (aod1)	0.859	1.754				
I believe social distancing has been suggested to help prevent infection of Covid-19 pandemic (aod2)	0.850	1.679				
I think tourism destinations plagued by the overpopulation of visitors (aod3)	0.829	1.753				
I would like to avoid overpopulated destinations because of Covid-19 (aod4)						
***Hygiene and safety***			0.803	0.810	0.883	0.716
After Covid-19, my need for hygiene while travelling is changed (hs1)	0.834	2.169				
I prefer destinations’ hygiene and cleanliness (hs2)	0.861	1.335				
I prefer destinations’ medical facilities (hs3)	0.870	1.432				
After Covid-19, I care more about the hygiene and safety of public transportation (hs4)	0.843	1.593				

Discriminant validity is the extent to which each latent variable is distinct from all other variables in the model [56]. Researchers [55] argued that the square root of the AVE for each variable should be higher than all of the relationships among the variable and other variables in the model. Table 2 showed the square roots of the AVE for the variables along the diagonal and the correlations among the indicators. The findings revealed that the square root of AVE is higher than all other values in the same row and column, which indicates that the model meets acceptable discriminant validity. We also considered the Heterotrait-Monotrait Ratio (HTMT) to estimate the discriminant validity of the model [65]. The results indicated that HTMT is lower than 0.90, which indicating that the discriminant validity meets the acceptable level [66].

**Table 2:** Discriminant validity.

	AOD	DIC	C19P	RM	SD	TRMP	TP	HS
Fornell-Larcker Criterion								
AOD	*0*.*846*							
DIC	0.394	*0*.*836*						
C19P	0.435	0.369	*0*.*831*					
RM	0.521	0.347	0.769	*0*.*826*				
SD	0.433	0.531	0.594	0.566	*0*.*880*			
TRMP	0.472	0.261	0.727	0.743	0.470	*0*.*842*		
TP	0.377	0.578	0.592	0.525	0.783	0.481	*0*.*873*	
HS	0.412	0.526	0.433	0.539	0.649	0.576	0.641	*0*.*877*
Heterotrait-Monotrait Ratio (HTMT)								
AOD								
DIC	0.489							
C19P	0.508	0.440						
RM	0.612	0.417	0.415					
SD	0.518	0.653	0.681	0.655				
TRMP	0.552	0.306	0.811	0.831	0.536			
TP	0.452	0.712	0.685	0.609	0.585	0.543		
HS	0.422	0.612	0.622	0.519	0.543	0.557	0.551	

*Note*: Covid-19 Pandemic (C19P), Travel risk and management perception (TRMP), Risk Management (RM), Service Delivery (SD), Transportation Patterns (TP), Distribution channels (DIC), Avoidance Overpopulated Destinations (AOD), Hygiene and safety (HS).

### Structural model analysis

The model’s predictive accuracy was estimated based on the explained variance portion (R^2^), whereas the R^2^ value of travel risk and management perceptions, risk management, service delivery, transportation patterns, distribution channels, avoidance of overpopulated destinations, and hygiene and safety were 0.628, 0.553, 0.521, 0.352, 0.668, 0.523, and 0.454 respectively. Based on [67], a non-parametric bootstrapping method was used to test the hypothesis relationships. The findings revealed that the effect of Covid-19 pandemic has significant impact on travel risk and management perceptions (β = 0.727, p < 0.01), and tourists’ travel risk and management perception has significant impact on risk management (β = 0.743, p < 0.01), service delivery (β = 0.470, p < 0.01), transportation patterns (β = 0.481, p < 0.01), distribution channels (β = 0.261, p < 0.01), avoidance overpopulated destinations (β = 0.472, p < 0.01), and hygiene and safety (β = 0.312, p < 0.01), thus, hypothesis H1-H7 are accepted (Table 3). The effect size was estimated using *f*^2^ values. Cohen (2013) [68] reported that *f*^2^ ≥ 0.02, *f*^2^ ≥ 0.15, and *f*^2^ ≥ 0.35 present small, medium, and large effect sizes respectively. The findings revealed that hygiene and safety (*f*^2^ = 0.365), transportation patterns (*f*^2^ = 0.356), and avoidance overpopulated destinations (*f*^2^ = 0.352) have a high effect size, whereas service delivery (*f*^2^ = 0.283), risk management (*f*^2^ = 0.236), and ravel risk perception (*f*^2^ = 0.356) have a medium effect size but distribution channels (*f*^2^ = 0.073) have a small effect size. The Q^2^ values for travel risk and management (0.349), risk management (0.350), service delivery (0.160), transportation pattern (0.166), distribution channel (0.036), avoidance of overpopulated destination (0.141), and hygiene and safety (0.132) were all larger than zero [69], indicating a predictive relevance of the construct.

**Table 3:** Path coefficients.

Hypothesis associations		Beta	SD	t-value	f^2^	Q^2^	R^2^	Decision
H1	C19P -> TRMP	0.727	0.042	17.471**	0.119	0.349	0.628	Accepted
H2	TRMP -> RM	0.743	0.041	18.215**	0.236	0.350	0.553	Accepted
H3	TRMP -> SD	0.470	0.078	6.022**	0.283	0.160	0.521	Accepted
H4	TRMP -> TP	0.481	0.074	6.540**	0.356	0.166	0.352	Accepted
H5	TRMP -> DIC	0.261	0.092	2.845**	0.073	0.036	0.668	Accepted
H6	TRMP -> AOD	0.472	0.074	6.374**	0.352	0.141	0.523	Accepted
H7	TRMP -> HS	0.312	0.068	4.588**	0.365	0.132	0.454	Accepted

Note: t-value ≥ 2.32 considers significant level at

**p<0.01 and t-value ≥ 1.64 considers significant level at

*p<0.05.

With respect mediating effects, the findings revealed that travel risk and management perception mediates the effect of Covid-19 pandemic on risk management (β = 0.540, t = 9.518, p < 0.01), service delivery (β = 0.341, t = 4.993, p < 0.01), transportation patterns (β = 0.350, t = 5.325, p < 0.01), distribution channels (β = 0.189, t = 2.688, p < 0.01), avoidance overpopulated destinations (β = 0.343, t = 5.612, p < 0.01), and hygiene and safety (β = 0.267, t = 3.869, p < 0.01), therefore H8a-H8f are accepted (Table 4).

**Table 4:** Mediating effects.

Hypothesis associations		Beta	SD	t-value	p-value	Decision
H8a	C19P -> TRMP -> RM	0.540	0.057	9.518	0.000	Accepted
H8b	C19P -> TRMP -> SD	0.341	0.068	4.993	0.000	Accepted
H8c	C19P -> TRMP -> TP	0.350	0.066	5.325	0.000	Accepted
H8d	C19P -> TRMP -> DIC	0.189	0.070	2.688	0.007	Accepted
H8e	C19P -> TRMP -> AOD	0.343	0.061	5.612	0.000	Accepted
H8f	C19P -> TRMP -> HS	0.267	0.069	3.869	0.000	Accepted

Note: C19P = Covid-19 Pandemic, TRMP = Travel risk and management perception, RM = Risk management, SV = Service delivery, TP = Transportation patterns, DIC = Distribution channels, AOD = Avoidance overpopulated destinations, Hygiene and safety (HS).

## Discussion

In this study, we aimed to evaluate the psychometric properties of the Covid-19 pandemic, a newly developed scale designed to measure the aspect of international tourists’ travel risk and management perceptions and its social outcomes. The results of the structural model assessment revealed the hypothesis relationships, which indicated that the Covid-19 pandemic has a relationship with travel risk and management perceptions. It implies that due to the spread of the Covid-19 pandemic across the globe, the majority of the countries were set up short-term travel limits to control the mass panic. By conducting a review of the previous study indicated that there is a relationship between perceived risk for disease-related factors and Covid-19 pandemic [13].

The existing study results identified that the effect of the Covid-19 pandemic has greatly affected risk management, service delivery, travel pattern, distribution channel, avoidance of overpopulated destinations, and hygiene and safety through the tourists’ travel risk and management perceptions. The tourists believe that Covid-19 pandemic has created travel risk and management perception and reduce their travel plant to destinations. Data analysis of this study specifies that tourists’ travel risk and management perception is greatly associated with risk management. In service research the Covid-19 pandemic context, risk management has been marked as a significant factor affecting an individual’s belief about controlling threats of a pandemic. The previous study [4] supported that tourists’ behavior can lead to risk management for destination infrastructure and medical facilities, destination image, and trip planning.

The result highlight that travels risk perception is associated with service delivery. This finding is related to [70] which found that there is a significant relationship between Covid-19 pandemic and service delivery. Tourists can avoid eating and drinking in restaurants. There is an alternative solution for people who can order delivery or takeout food to minimize interpersonal interaction. This study expands the existing knowledge by examining the effect of travel risk and management perception on travel pattern. This result is related to [4] who reported that travel pattern can lead to independent travel or small group tours, less group dining, promote destinations experiencing under tourism, and diversity such as novel outdoor activities, smart tourism, and nature-based travel. The findings indicated there is a positive association between travel risk and management perception and distribution channels. It infers that distribution Chanel can encourage people for nature-based travel and smart tourism to reduce the travel risk and risk management perception during the Covid-19 pandemic. some researchers have reported that people can use technology for travel-related purposes to reduce travel risk and risk management perception [9].

The empirical results indicated that tourists’ travel risk and management perception is greatly associated with the avoidance of overpopulated destinations. The effect of Covid-19 pandemic spreads through human-to-human transmission, thus, avoidance of overcrowded destinations can be an alternative solution to reduce infection [44]. The overpopulated destinations can be minimized by using a short-term strategy of imposing travel restrictions for certain attractions destinations. Data analysis point out that the travel risk and management perception have a positive impact on hygiene and safety, which corresponds well with a previous study [4] which indicated that travel risk and management perception has greatly affected tourists’ travel decision and their perceptions of hygiene and safety due to the spread of Covid-19 epidemic. In the context of service research, hygiene and safety judgments have been marked as an important construct affecting people’s safety and security towards the service firm or customers’ purchase intention of goods and services offered by the firms or service organizations. Tourists can purchase travel insurance when booking trips to confirm coverage in case of illness including Covid-19. Usually, the potential tourists are likely to express their interest in destinations’ hygiene, safety, security, cleanliness, avoidance population density, and medical facilities when they decide for travelling to destinations.

### Implications

The findings of this study indicated that Covid-19 has affected tourists’ travel risk and management perceptions and its impact on risk management, service delivery, transportation patterns, distribution channels, avoidance of overpopulated destinations, hygiene and safety. Tourists believe that Covid-19 pandemic has created tourists’ health anxiety and reduce their travel plans for destinations. These findings may help policy-makers and healthcare operators to manage maladaptive levels of concern due to Covid-19 pandemic, and to know who is more inclined to react unpleasantly towards the Covid-19 pandemic. Health practitioners can improve educational interventions while targeting international tourists for travel destinations. Tourists are worried about the spread of Covid-19 pandemic on their travel activities and travel-related preferences in the post-pandemic period. With the significant effect of Covid-19 pandemic, this study contributes key insights to assist tourism policymakers and practitioners improve effective strategies to enhance tourists’ confidence after facing health risk crisis and travel risk and management perception towards travel destinations. The travel movement has become more selective, therefore independent travel and health tourism are crucial. Tourists can take fewer trips but spend longer in their picked destinations. These patterns will reduce the negative effects of the travel industry and lessen tourists’ travel risk and management perceptions. Based on the tourists’ travel risk and management perceptions and travel recuperation systems, travel attributes can move in the present due to the spread of Covid-19 epidemic.

The disaster of Covid-19 pandemic teaches us not to visit overpopulated destinations and those people suffering from overcrowded destinations, there is a necessity to evaluate their travel planning and improvement to ensure sustainability. As tourists prefer quiet destinations for their tourism activities due to the Covid-19 pandemic, the global travel and tourism industry could benefit by paying attention to these craving. Due to these predicted changes in tourist behavior, the world tourism industry entails close academic attention. The travel and tourism industry is a fundamental part of the global economy, liable for a large number of occupations and billions of dollars in profit. Therefore, travel and tourism industry practitioners and policymakers should reevaluate tourists’ behavior, travel industry policies, regulations, tourism operators’ market, and tourism product development to promote continuous sustainability. The existing global health crisis has an unprecedented impact on the travel and tourism industry due to the spread of Covid-19 pandemic.

Tourists’ travel risk and management perceptions and their impacts on the tourism market or society (e.g. risk management perception, service delivery, transportation patterns, distribution Channels, avoidance of overpopulated destinations, hygiene and safety), need a top to bottom investigation to empower the tourism industry experts, and policymakers to build up a more adjusted industry. Tourists’ travel risk and management perceptions in the tourism industry will likewise prompt the development of new tourism markets that academics and tourism operators can investigate together. The findings of the existing empirical study are likely to shape theories on tourists’ travel risk and management perceptions, tourists’ behavior, marketing and management, both in the travel and tourism industry explicitly and in more extensive fields in general. The spread of Covid-19 flare-up has carried critical effects on society and industry. The travel and tourism policymakers and academicians should consider this pandemic tragedy and how it will advise tourism industry practices. The potential tourists concern about how they travel to destinations; thus, tourism practitioners should consider the strategies that mitigate the spread of a pandemic, public health crises, and ponder a plan that carries positive changes to the travel industry following this pandemic. For example, tourists should be needed to buy travel insurance when booking trips to guarantee coverage if there should be an occurrence of sickness, including a post-covid pandemic. Both international and domestic tourism needs to stress safety and health measures, and any tourism activities that make tourists feel safer to travel destinations and reduce their travel risk and management perception. The impact of Covid-19 pandemic should be considered within a global community. The spread of Covid-19 epidemic will have greater psychological, sociological and financial impacts if it is not eliminated quickly across the world. While society can recuperate effectively from financial interruption, including in global travel and tourism activities, following Covid-19 pandemic, the sociological and mental effects will be more stable. People should explore the current post-covid pandemic scene cautiously and sympathetically.

## Limitation and future study

This study has several limitations despite its strengths such as large sample size and a relatively heterogeneous sample of the international tourists who visited the destination for leisure/holiday or shopping purposes, education/conference, healthcare, business and other purposes. This study surveyed with self-administrative questionnaire report measures that entail potential bias assumes that participants might be influenced by social desirability. Therefore, future study should aim to use other measures such as opinions of focus groups, which could support more in-depth analysis. This study employed a quantitative method that is inflexible to participants’ subjective views on the effect of Covid-19 pandemic, thus, future study is suggested to ask qualitative assessments using in-depth interviews. The data was collected through the online platform, which much easier for the young generations compare to the older generations, and leads to a large number of a younger group of participants. A limited number of items were used to evaluate the constructs of the conceptual model and thus future studies should cover the large measurement items. The objective of this study mainly focuses on the impact of Covid-19 pandemic on tourist travel risk and management perception to assist the tourism industry to provide coping strategies in the face of the tourism crisis. Thus, future study should be conducted to investigate the factors that influencing tourists travel risk attitudes and risk management perceptions during and after the Covid-19 epidemic. This might be helpful for tourism managers and practitioners to pay attention to the control of Covid-19 crisis, and a systematic management strategy to promote the development of the tourism industry.

## Supporting information

S1 File (CSV)

S1 Dataset (XLSX)

S2 Dataset (XLSX)
